# Supplementary material for: Single-cell RNA sequencing reveals the mesangial identity and species diversity of glomerular cell transcriptomes
Source: Nat Commun. 2021 Apr 9;12:2141. doi: 10.1038/s41467-021-22331-9 (PMC8035407; doi:10.1038/s41467-021-22331-9)
Supplement: Supplementary file 1 — Supplementary Information [file 41467_2021_22331_MOESM1_ESM.pdf]

Supplementary Figure 1

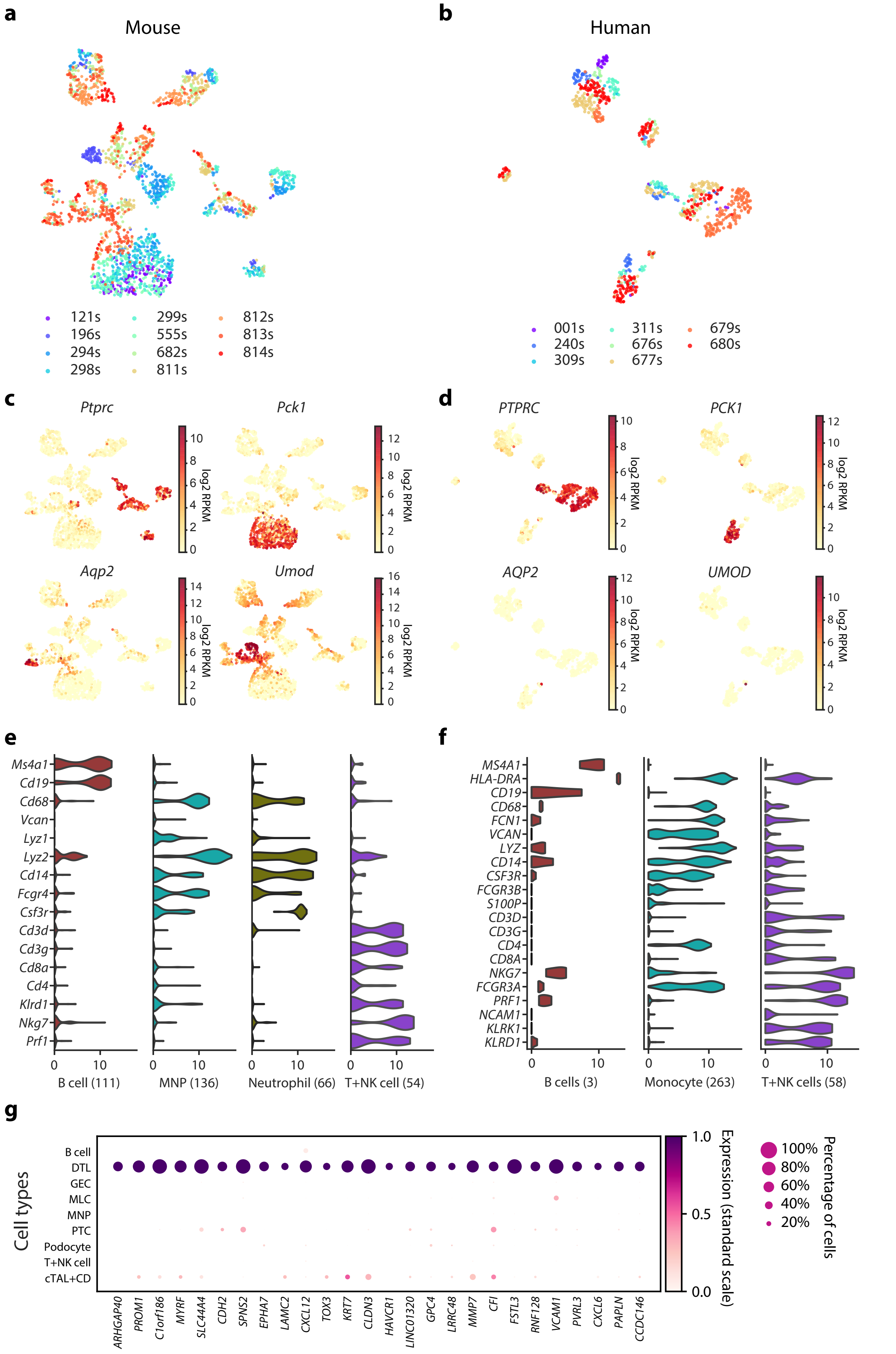

Supplementary Figure 1. Experimental batch/donor information and captured non-glomerular cell populations. a-b Projection of C57BL/6J mouse and human glomerular associated single cells onto 2-D UMAP space coloured by Smart-seq2 plate ID numbers. **c-d** The expression (log2-RPKM) of non-glomerular cell type markers in mouse and human such as *Ptprc* for leukocyte; *Pck1* for proximal tubular cells; *Aqp2* for collecting ductal cells; *Umod* for cortical thick ascending limb of the loop of Henle (cTAL). The colour scale is defined by log2(mean RPKM). **e-f** The expression (log2-RPKM) of immune cell type markers in identified mouse and human immune cell subpopulations. **g** The expression of 26 significant up-regulated genes in human DTL cells. The colour intensity and size of each dot represents the mean expression (standard scale) and the percentage of cells expressing each gene in individual cell types, respectively.

Supplementary Figure 2

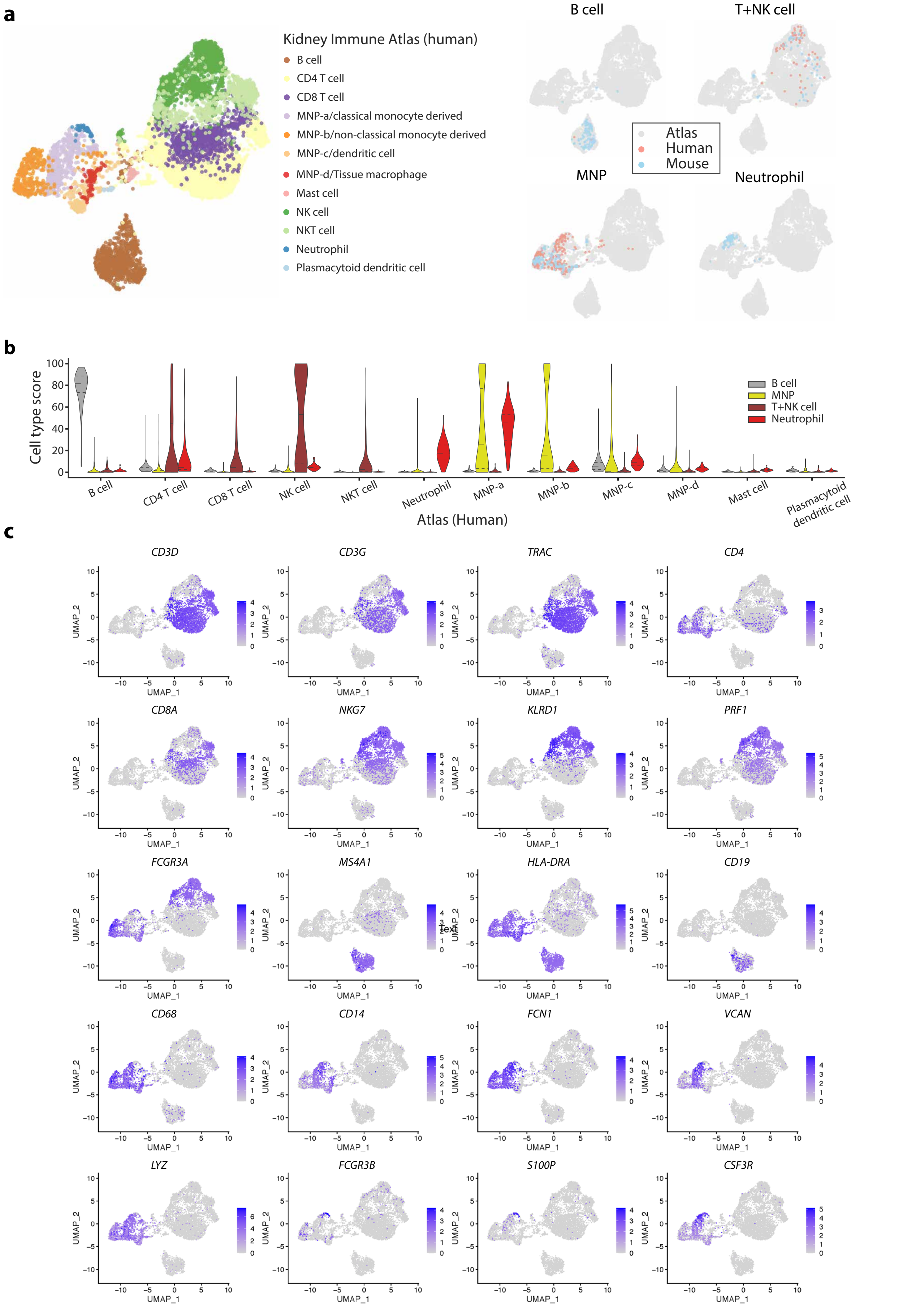

Supplementary Figure 2. Single-cell data integration of mouse and human glomerulus-associated immune cells with data of the human kidney immune cell atlas. a Projection of the human kidney immune cell atlas[17] (left), mouse and human glomerulus-associated immune cells (right) onto 2-D UMAP space from multiple dataset integration (Seurat v3). **b** Cell type prediction of mouse and human glomerulus-associated immune cells using the human kidney immune cell atlas as a reference. Cells were grouped by assigned cell types from clustering. **c** The expression of immune cell type markers in integrated single cell data.

Supplementary Figure 3

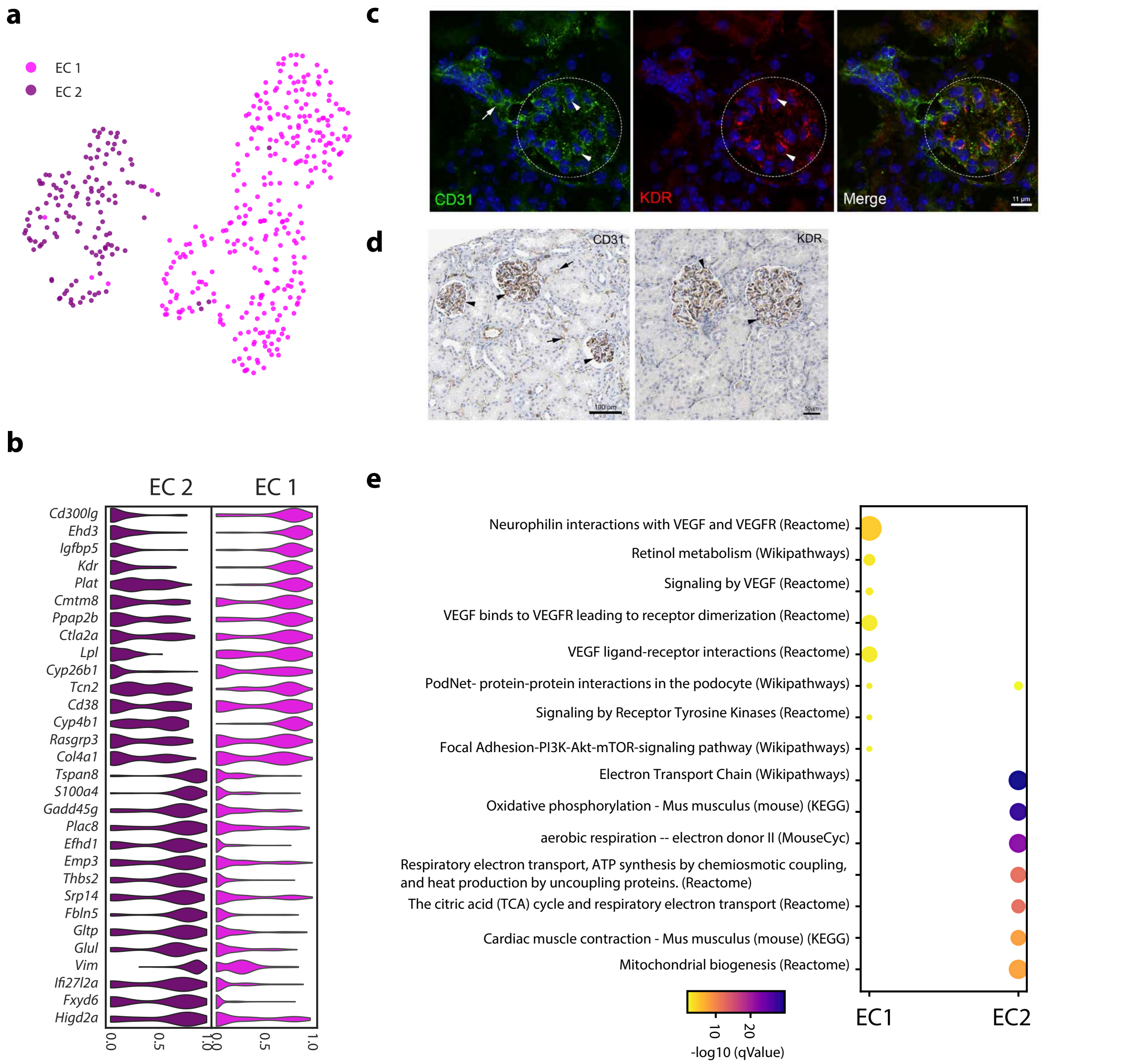

Supplementary Figure 3. The transcriptome profiling of mouse glomerular endothelial cells (GECs). a UMAP projection of mouse glomerulus-associated endothelial cells onto 2-D UMAP space coloured by endothelial subclusters (EC1 and EC2). **b** Violin plots showing the expression of top 15 genes significantly upregulated in each endothelial subcluster (EC1 and EC2). The genes were selected based on the magnitude of expression range between EC1 and EC2. The full list of differentially expressed genes is presented in Supplementary Data 1. **c** Double labelling of CD31 (green) and KDR (also called VEGFR2) (red) shows KDR present only in mouse GECs (arrowheads), whereas CD31 is detected in both GECs and extraglomerular ECs (arrows). Discontinuous circles indicate glomeruli. Scale bar: 11 μm. **d** Immunohistochemistry for CD31 and KDR in human kidney tissues shows that KDR is abundantly and specifically expressed in GECs (arrowheads), whereas CD31 is expressed in both GECs (arrowheads) and extraglomerular ECs (arrows). Images are downloaded from www.proteinatlas.org (https://creativecommons.org/licenses/by-sa/3.0/). Scale bars are indicated in images. **e** Top enriched pathways of genes significantly upregulated in each endothelial subcluster. The dot colour indicates the pathway significance based on -log10(qValue) from FDR multiple testing. The dot size shows the percentage of genes in each pathway gene set were significantly upregulated in EC1 or EC2, in which the smallest and largest dot on the plot represent the percentage of 2 and 75 respectively. The full list of enriched pathways is shown in Supplementary data 2-S3.

Supplementary Figure 4

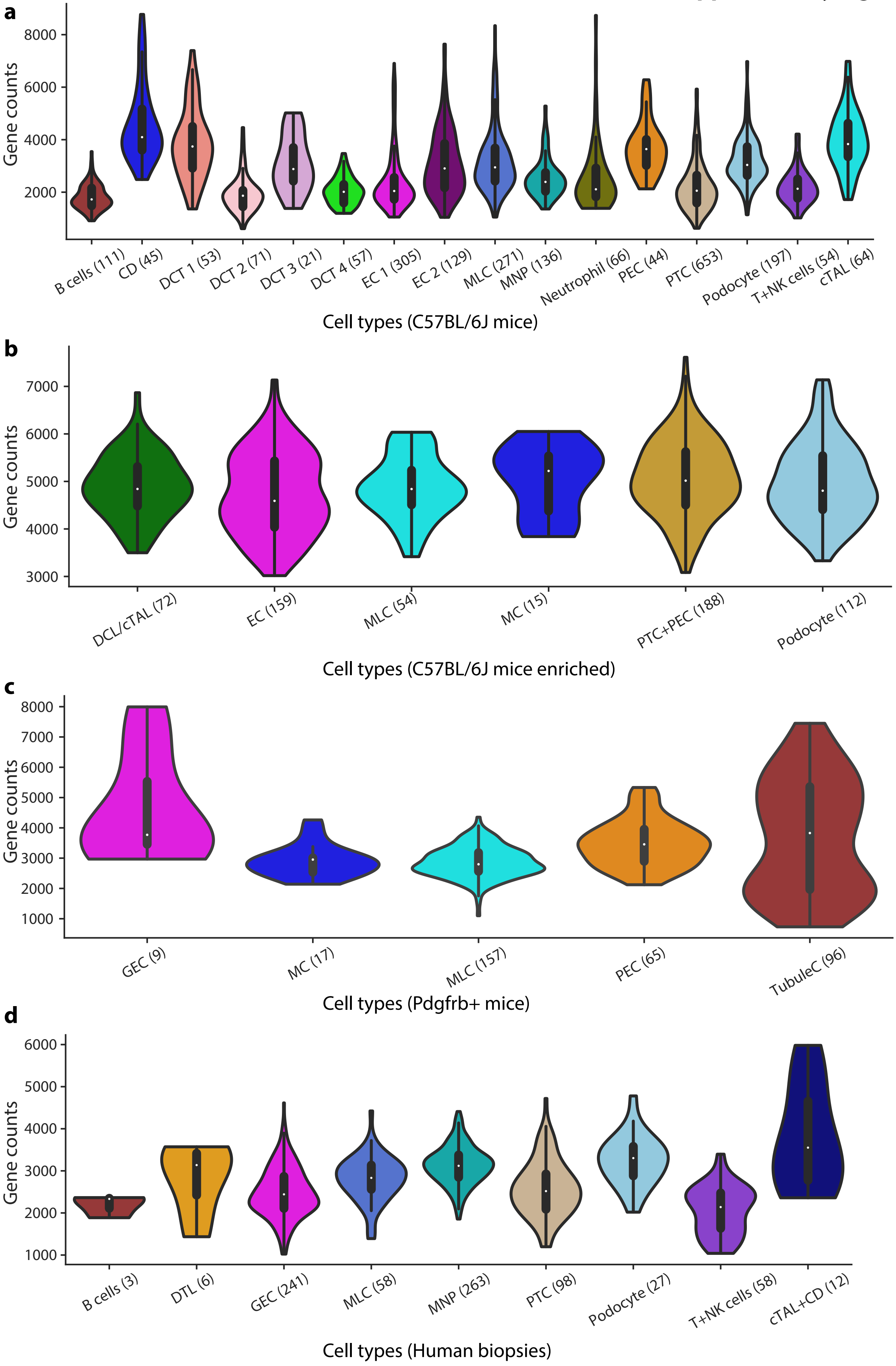

Supplementary Figure 4. The number of genes detected per cell and per cell type from mouse and human glomerulus-associated single cell cohorts in this study. **a** C57BL/6J mouse single cells using the unbiased sorting strategy. **b** C57BL/6J mouse enriched glomerular single cells using the CD45$^-$ CD31$^-$ sorting strategy. **c** Pdgfrb$^+$ mouse glomerulus-associated single cells by sorting EGFP$^+$ cells from *Pdgfrb*-EGFP reporter mice. **d** Human kidney biopsy single cells using the unbiased sorting strategy. Genes were defined as detected in a cell when RPKM > 1.

Supplementary Figure 5

a

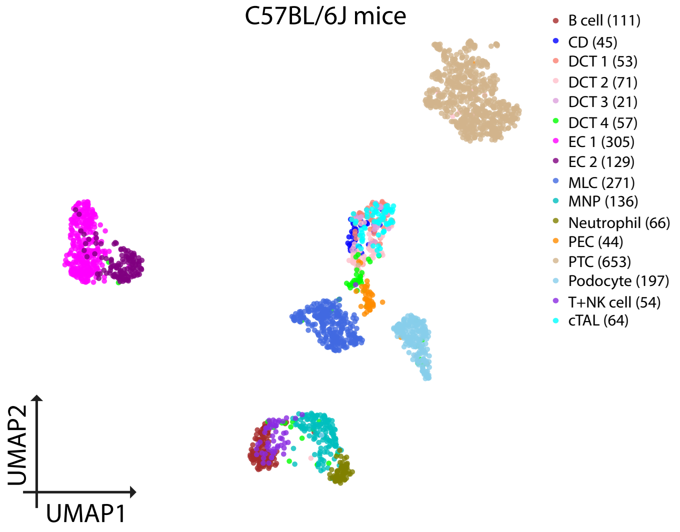

b

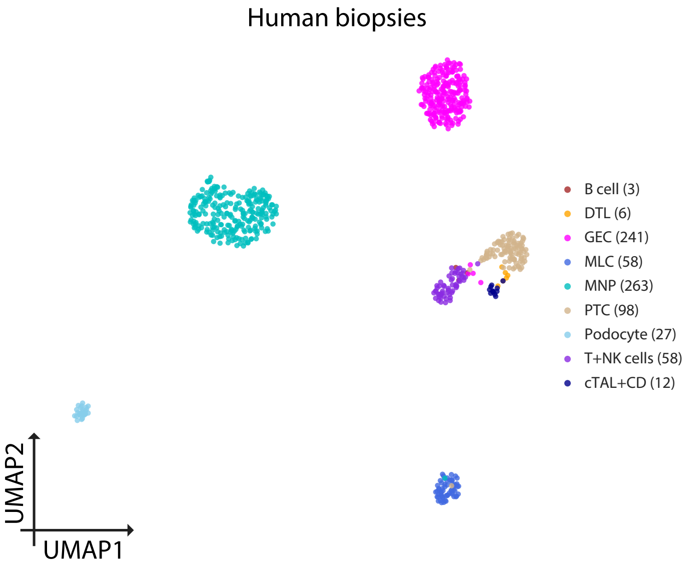

Supplementary Figure 5. UMAP trajectory of mouse and human single cells based on binary regulon activity scores. a The single cells from C57BL/6J mice. **b** The single cells from human kidney biopsies.

Supplementary Figure 6

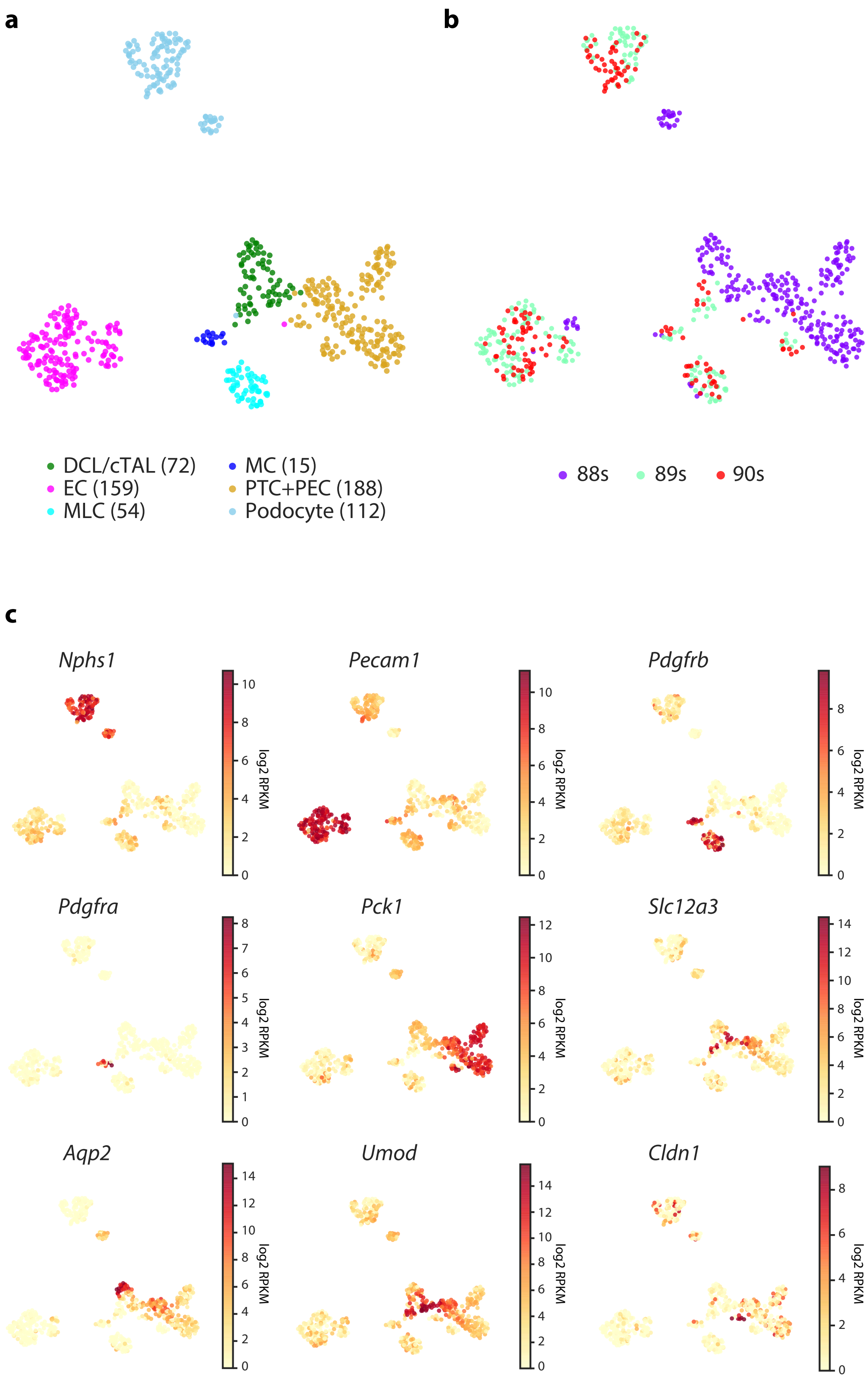

Supplementary Figure 6. scRNA-seq analysis of mouse enriched glomerular cells by $CD45^-$ $CD31^-$ sorting. a-b UMAP visualization of enriched mouse (C57BL/6J) single cells coloured by cell type annotation (left) and batch number (right). **c** The expression of selected cell type markers. No $CD45^+$ immune cells are detected, but $CD31^+$ ECs are still present. The colour scale is defined by log2(mean RPKM).

Supplementary Figure 7

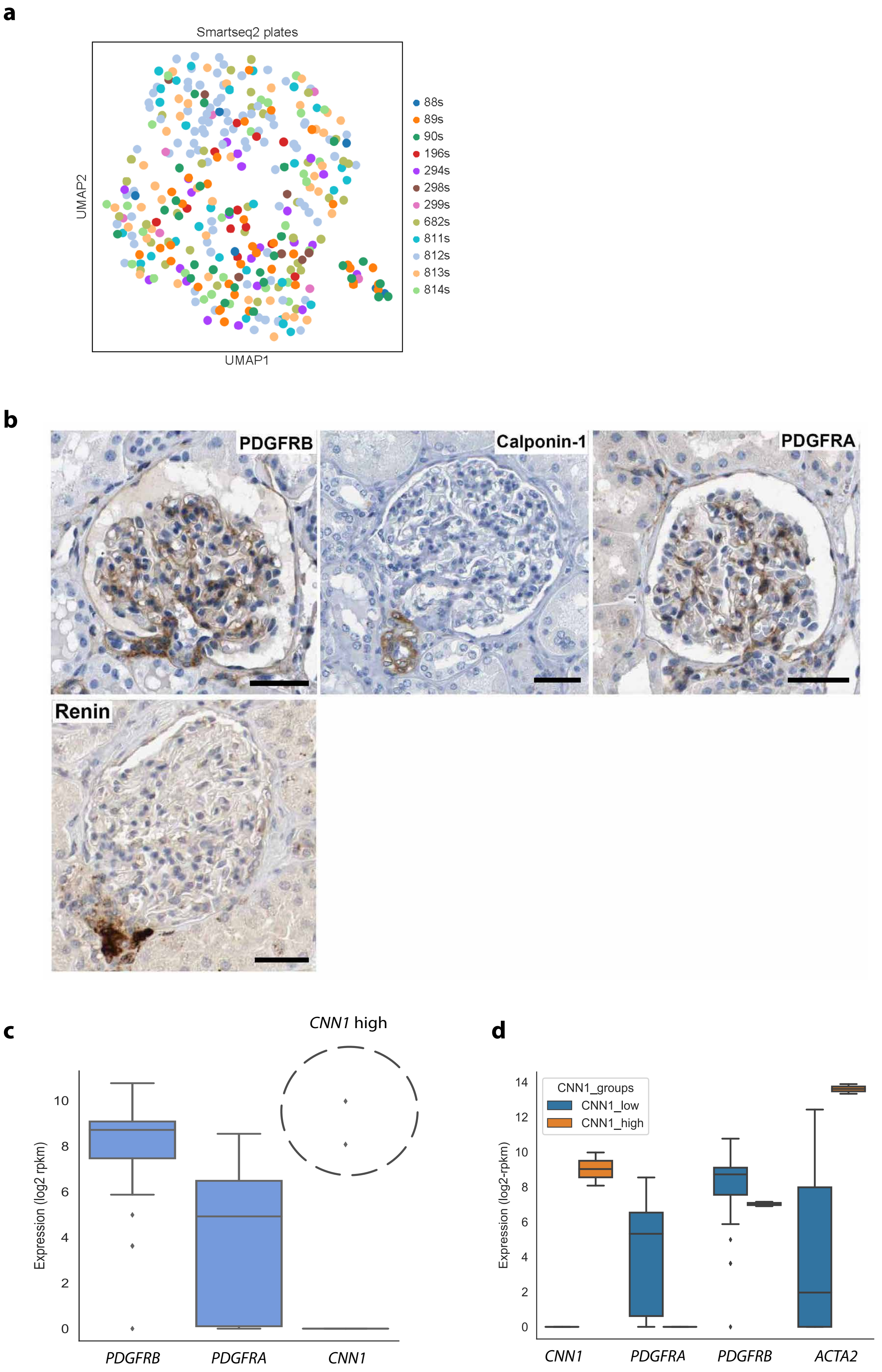

Supplementary Figure 7. Characterization of MLC heterogeneity. a UMAP projection of combined mouse MLCs from two datasets by unbiased and $CD45^-$ $CD31^-$ sorting onto 2-D UMAP space coloured by Smart-seq2 plate ID numbers. **b** Immunohistochemistry of PDGFRB, calponin-1 encoded by *CNN1*, PDGFRA and renin in human kidney. PDGFRB is positive in intraglomerular MCs and vSMCs; calponin-1 is only expressed in vSMCs; PDGFRA is detected in intraglomerular MCs but not in vSMCs. Image data are downloaded from proteinatlas.org (https://creativecommons.org/licenses/by-sa/3.0/). Scale bars for all: 50 µm. **c** The expression (log2-RPKM) of *PDGFRB*, *PDGFRA* and *CNN1* in human MLCs. Only two cells (circle) expressed *CNN1* at the high level. **d** The expression (log2-RPKM) of *CNN1*, *PDGFRA*, *PDGFRB* and *ACTA2* in human MLCs grouped by *CNN1* high (n=2) and low (n=56) cells. The two *CNN1*-highly expressing cells are vSMCs and the remaining cells are intraglomerular MCs. Magnifications: B, x200.

Supplementary Figure 8

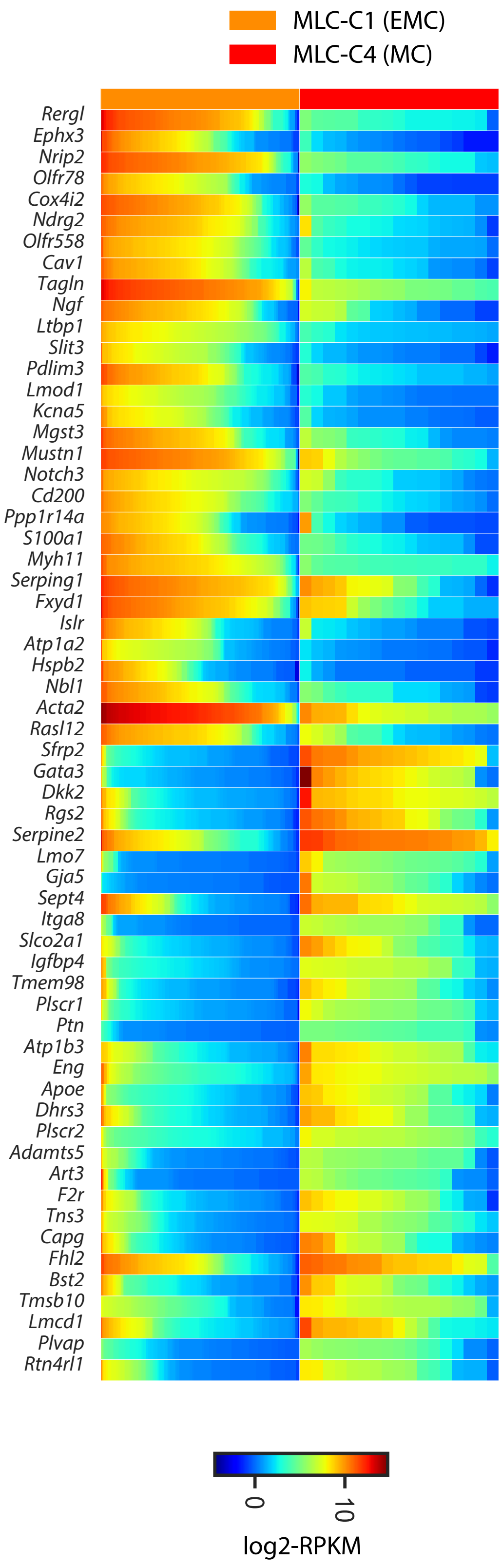

Supplementary Figure 8. Comparison of MLC-C1 (EMCs) and MLC-C4 (intraglomerular MCs) populations. Heatmap for top genes differentially expressed between mouse MLC-C1 and MLC-C4 cell clusters. The full list of differentially expressed genes is presented in Supplementary Data 6.

Supplementary Figure 9

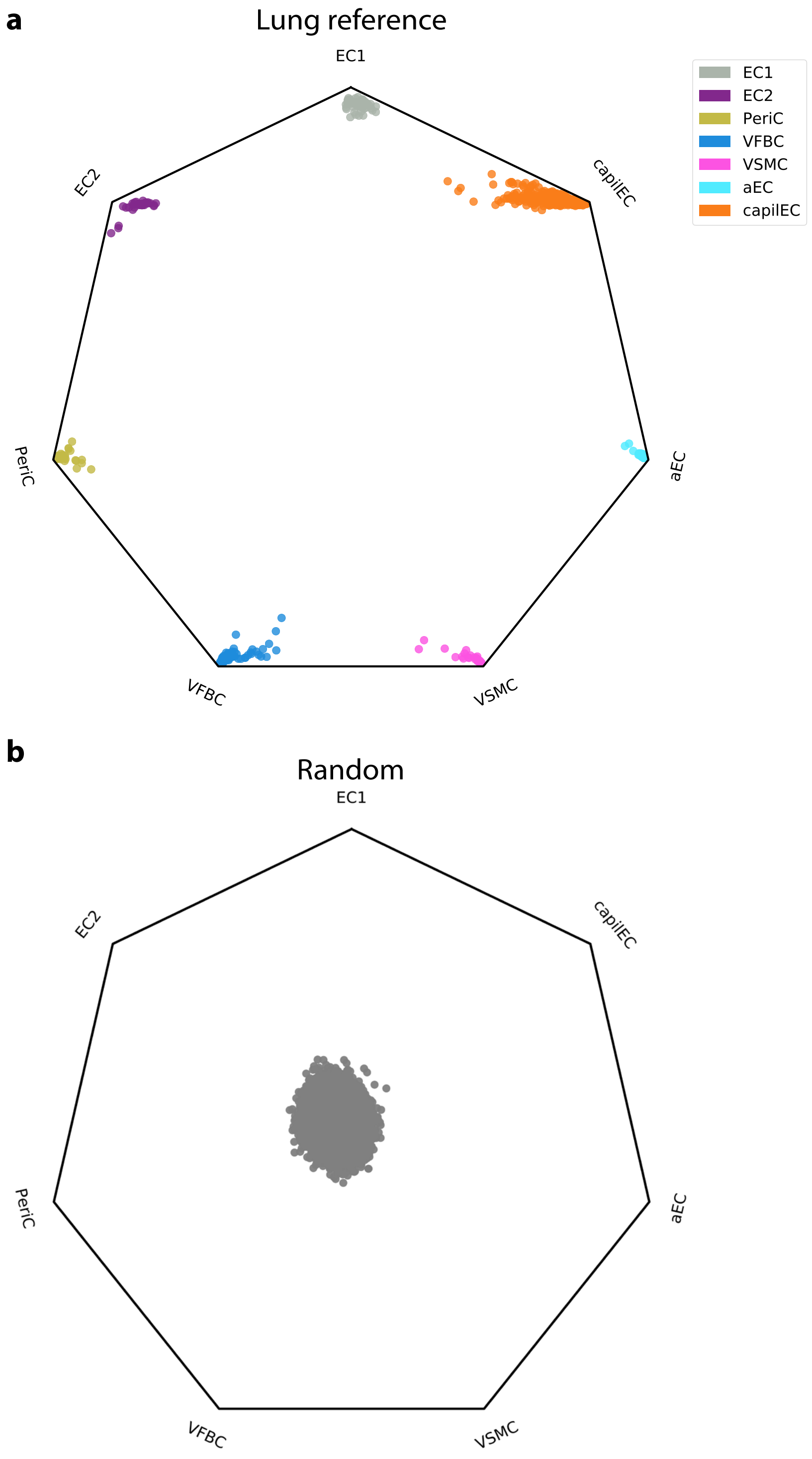

Supplementary Figure 9. Radar visualization of the probabilistic similarity of reference cells and negative control cells. a Reference cells show high similarity to their respective cell types. **b** Negative control cells obtained from randomized transcriptional values did not show any significant similarity to any of the cell types.

Supplementary Figure 10

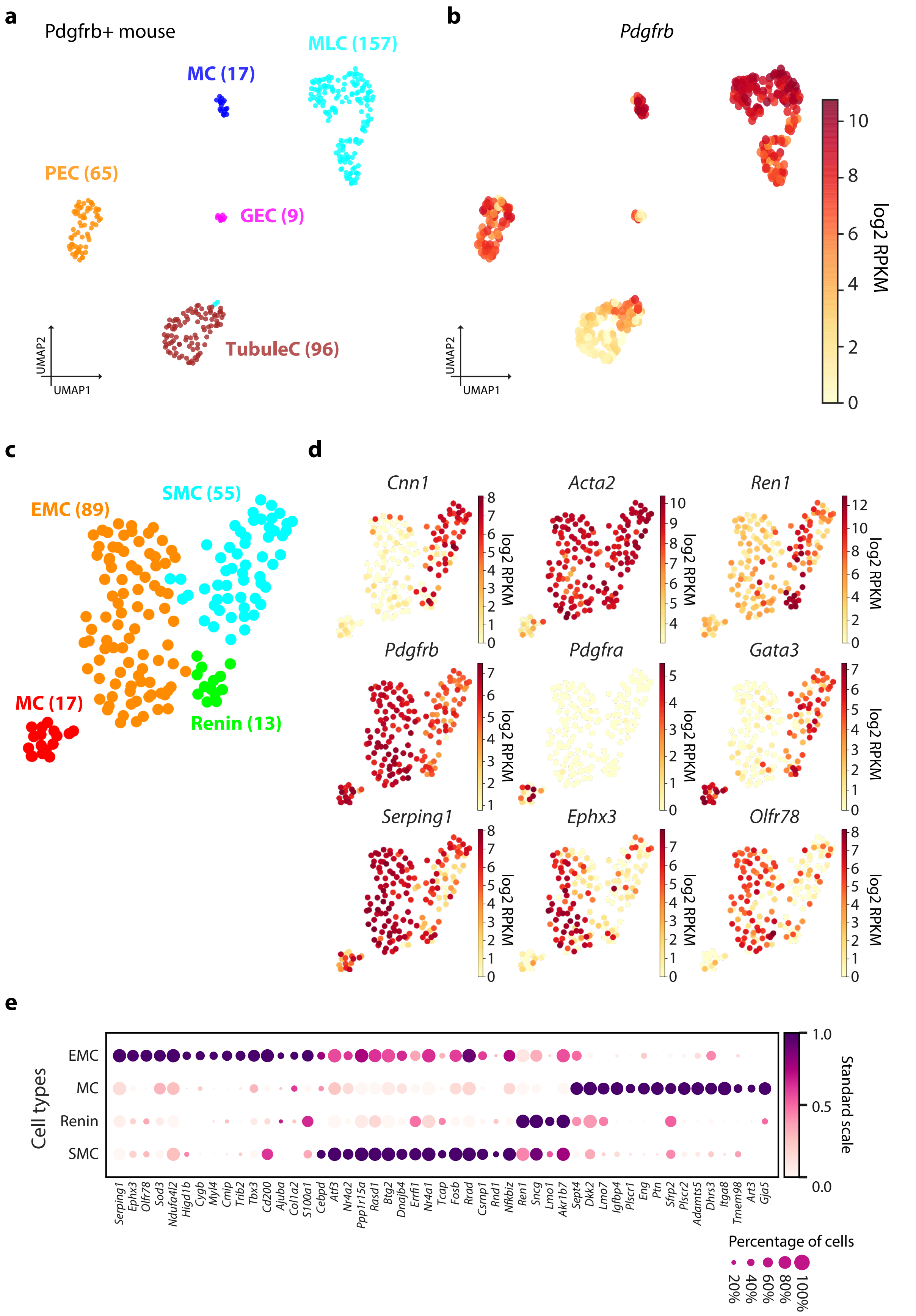

Suppelementary Figure 10. Validation of MLC subpopulations using single cells from *Pdgfrb*-EGFP reporter mice. a-b UMAP of isolated glomerular $Pdgfrb^+$ single cells by $EGFP^+$ cell sorting from *Pdgfrb*-EGFP reporter mice coloured by assigned cell types (left) and the expression of *Pdgfrb*. The cell number of cell clusters is presented in parenthesis. The colour scale is defined by log2(mean RPKM). MC: mesangial cell, MLC: mesangial-like cell, PEC: glomerular parietal epithelial cell, GEC: glomerular endothelial cell, TubuleC: tubular cell. **c** Re-clustering of MLCs from $Pdgfrb^+$ mice showing four subpopulations annotated as EMC, vSMC, renin cell and MC. The cell number of four subpopulations is presented in parenthesis. **d** The expression of vSMC, renin cell, MC and EMC markers in single cell populations. **e** The expression of top MLC cell type signatures obtained from C57BL/6J mice (shown in Fig. 2f) in single cells from *Pdgfrb*-EGFP reporter mice. The colour intensity and size of each dot represent the mean expression (standard scale) and the percentage of cells expressing each gene (x-axis) in individual cell types (y-axis), respectively.

Supplementary Figure 11

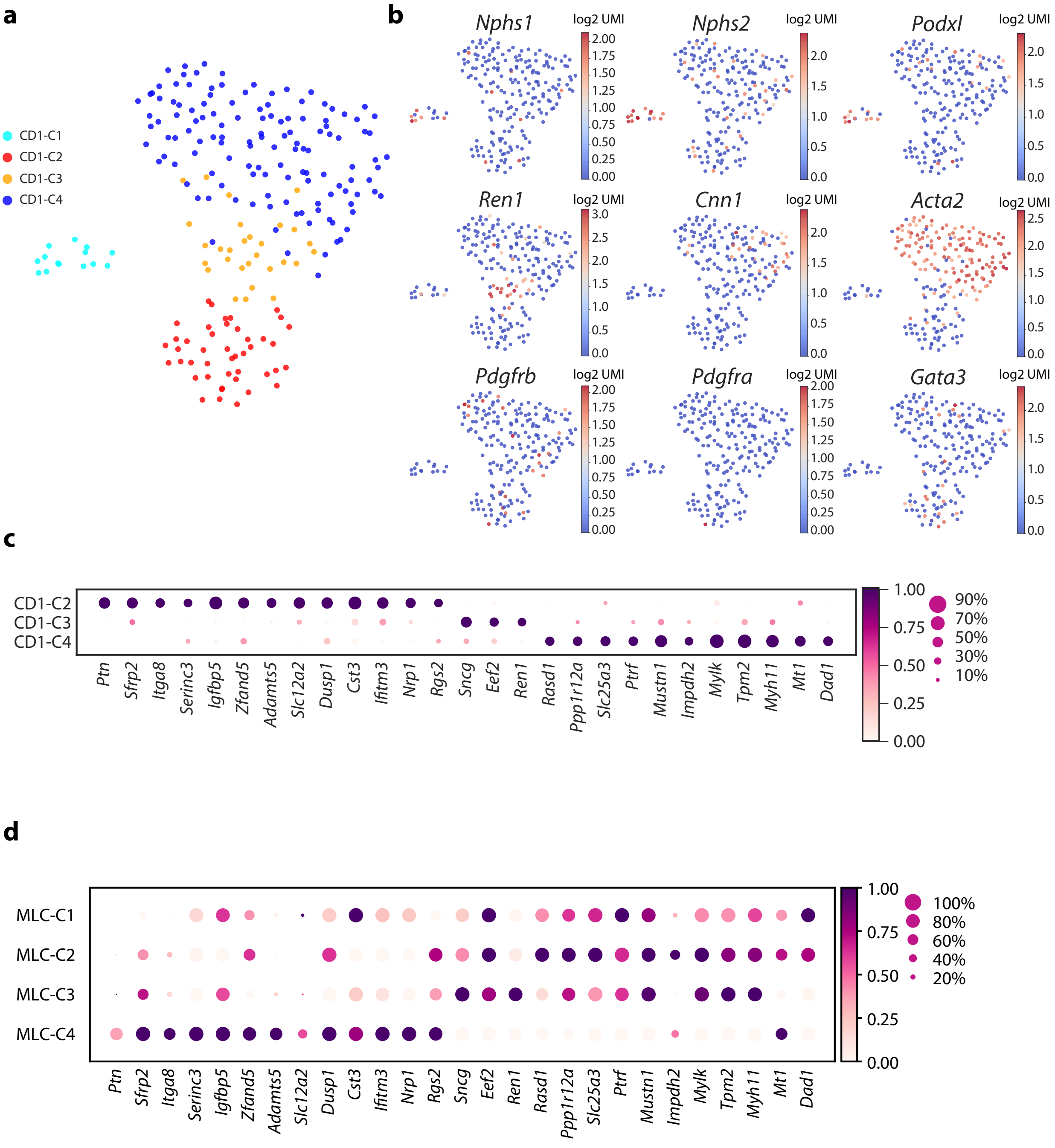

Supplementary Figure 11. Re-analysis of MCs from published scRNA-seq data[5] shows four distinct subpopulations. a-b UMAP projection of all pre-defined MCs from isolated CD1-mouse glomeruli[5] onto 2-D UMAP space coloured by subclusters and by cell type marker expression (log2-UMI counts). CD1-C1, CD1-C2, CD1-C3 and CD1-C4 correspond to contaminated cell with podocyte signature, true mesangial cells, renin cells and vSMCs, respectively. **c-d** The expression of significant differentially expressed genes from the comparison of CD1-C2 vs. CD1-C3 vs. CD1-C4 in published data from CD1 mice (**c**) and in data from our C57BL/6J mice (**d**). The colour intensity and size of each dot represents the mean expression (standard scale) and the percentage of cells expressing each gene in each subpopulation, respectively. CD1-C2 cells are probably genuine MCs as genes significantly overexpressed in CD1-C2 are enriched also in MLC-C4. CD1-C3 and CD1-C4 most likely correspond to MLC-C2 and MLC-C3 subpopulations in our study.

Supplementary Figure 12

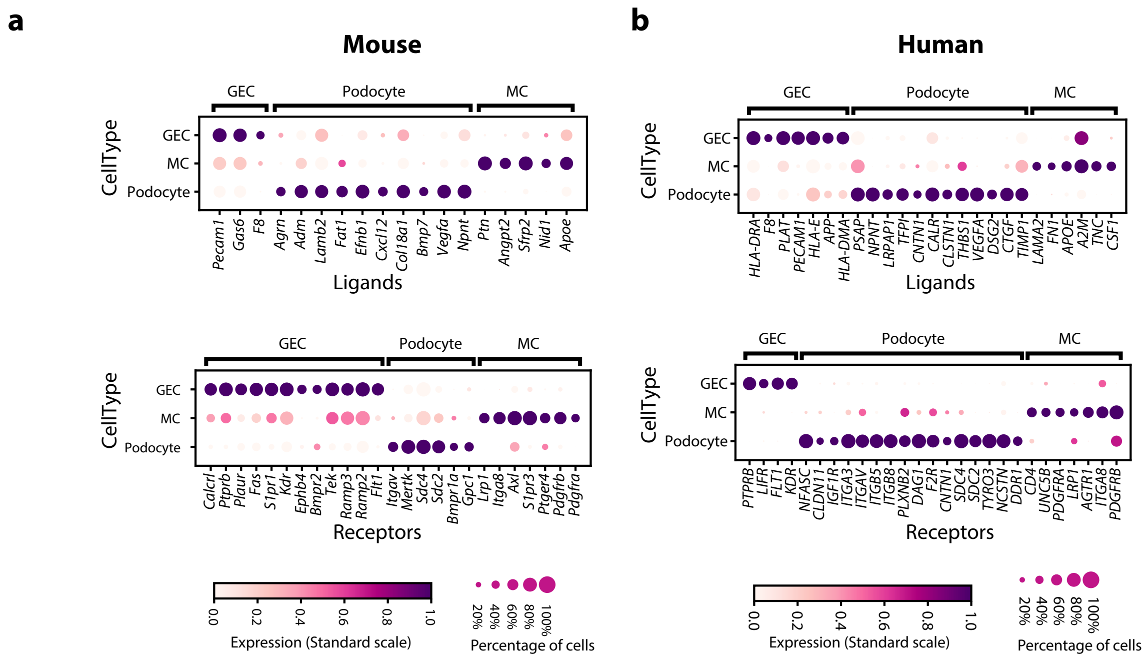

Supplementary Figure 12. Expression of glomerular cell type specific ligands and receptors in mouse and human. The colour intensity and size of each dot represent the mean expression (standard scale) and the percentage of cells expressing each gene (x-axis) in individual cell types (y-axis), respectively. **a** Mouse data. **b** Human data.

Supplementary Figure 13

a

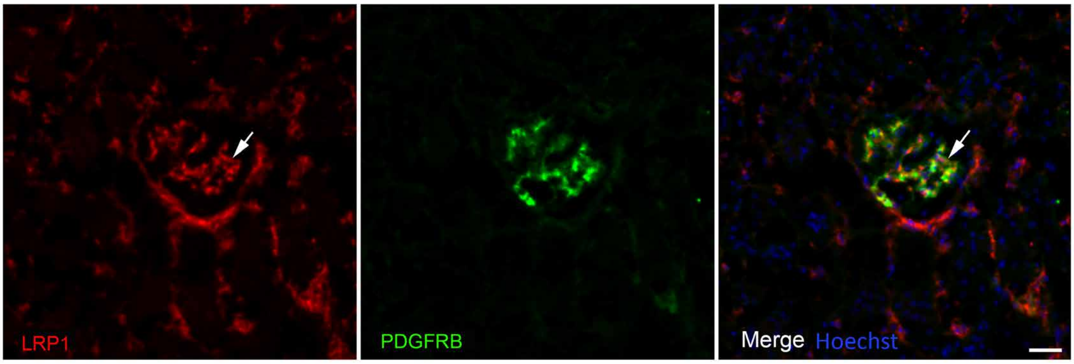

b

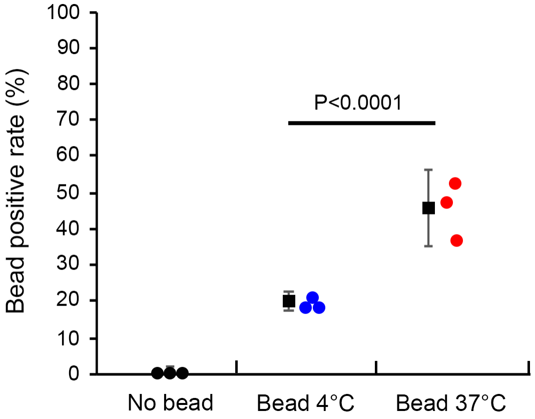

Supplementary Figure 13. Expression of LDL receptor related protein 1 (LRP1) in mesangial cells and phagocytosis by mouse blood leukocytes. **a** Immunostaining for LRP1 in the mouse glomerulus. Signal for LRP1 (red) is visible in PDGFRB-labelled (green) MCs (arrow). Nuclei (blue) was stained by the Hoechst dye. Scale bar: 30 μm. **b** Phagocytosis by mouse blood leukocytes. Phagocytosis of conjugated latex bead by mouse peripheral blood leukocytes was analysed using FACS analysis. Dots represent percentages (%) of bead positive cells in triplicate independent assays in three groups (no beads 4°C, beads 4°C and beads 37°C). Each assay included two mice. Error bars are defined as mean values and SD. The two-sided P value of 2.2×10^{-16} was calculated using the proportion test. All original flow cytometry plots and gating raw data are available in the Source data file.

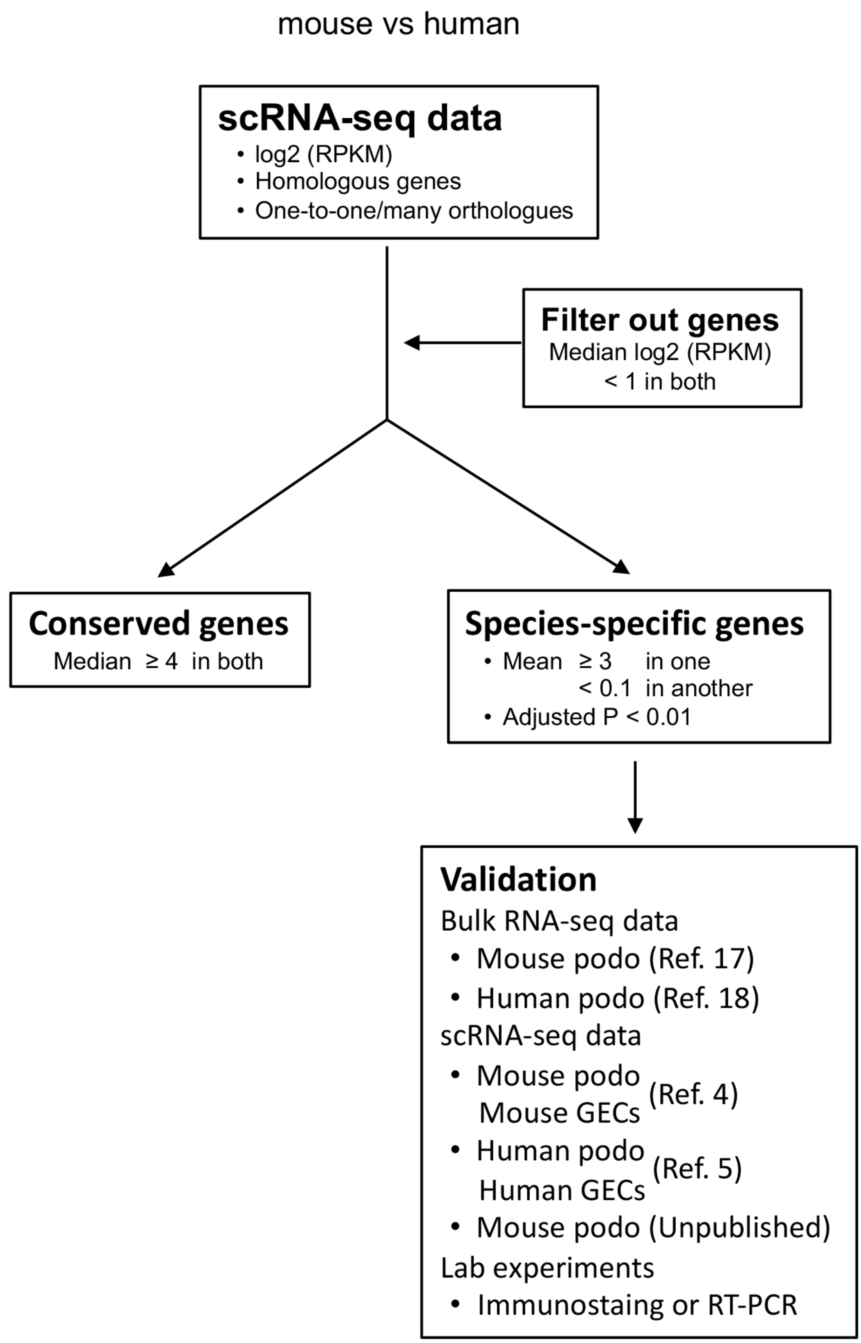
mouse vs human
scRNA-seq data
• log2 (RPKM)
• Homologous genes
• One-to-one/many orthologues
Filter out genes
Median log2 (RPKM)
< 1 in both
Conserved genes
Median ≥ 4 in both
Species-specific genes
• Mean ≥ 3 in one
< 0.1 in another
• Adjusted P < 0.01
Validation
Bulk RNA-seq data
• Mouse podo (Ref. 17)
• Human podo (Ref. 18)
scRNA-seq data
• Mouse podo
Mouse GECs
(Ref. 4)
• Human podo
Human GECs
(Ref. 5)
• Mouse podo (Unpublished)
Lab experiments
• Immunostaing or RT-PCR

Supplementary Figure 14. Workflow of species comparison of three principal glomerular cell transcriptomes.

Supplementary Figure 15

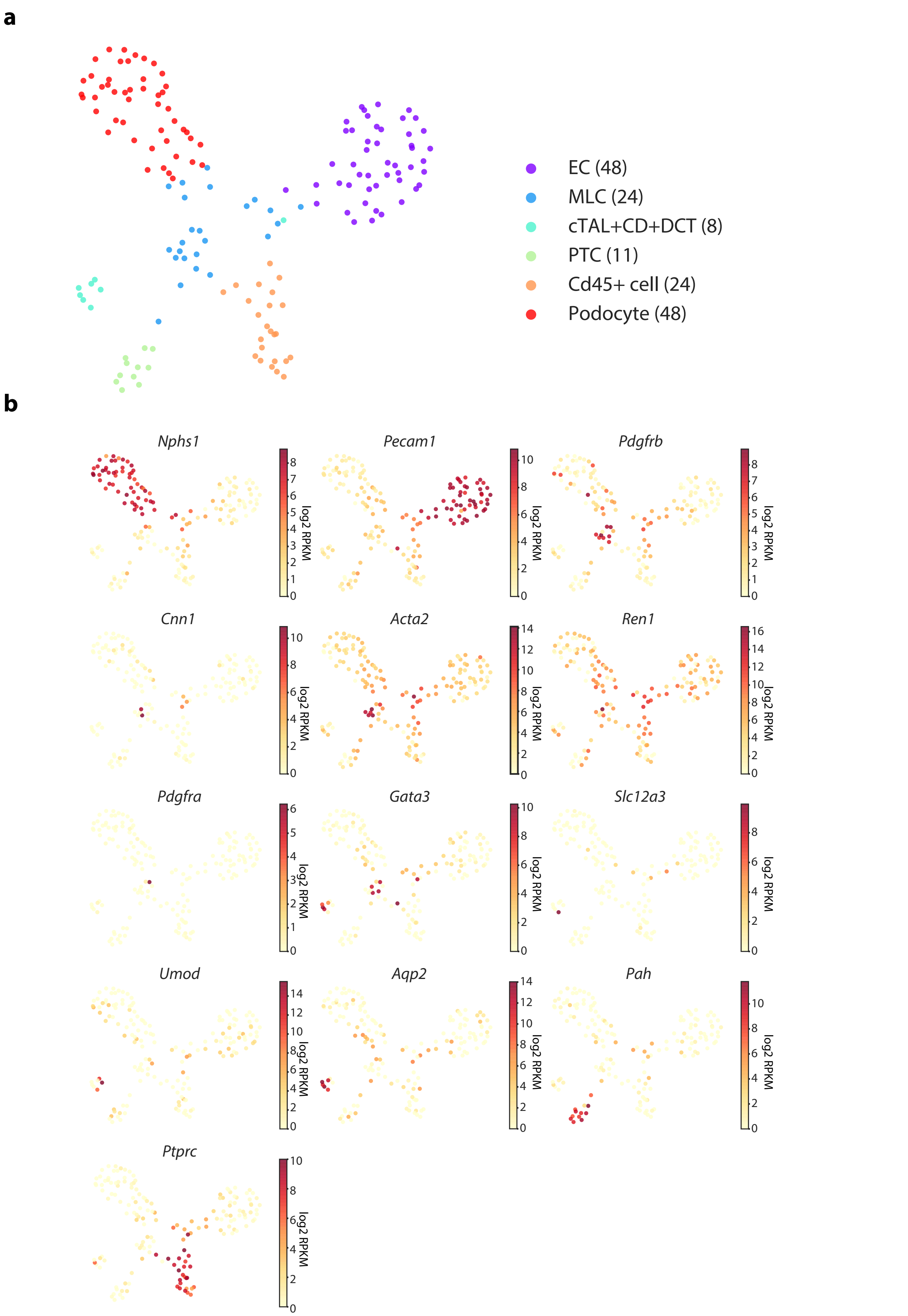

Supplementary Figure 15. scRNA-seq analysis of mouse glomerulus-associated single cells using the bead-free method isolating mouse glomeruli. a UMAP of single cells coloured by assigned cell types. EC: endothelial cell, MLC: mesangial-like cell, cTAL: cortical thick ascending limb, CD: collecting duct, DCT: distal convoluted tubule, PTC: proximal tubule cell. The cell number of clusters is presented in parenthesis. **b** UMAP coloured by the expression of cell type markers. The colour scale is defined by log2(mean RPKM).

Supplementary Figure 16

a

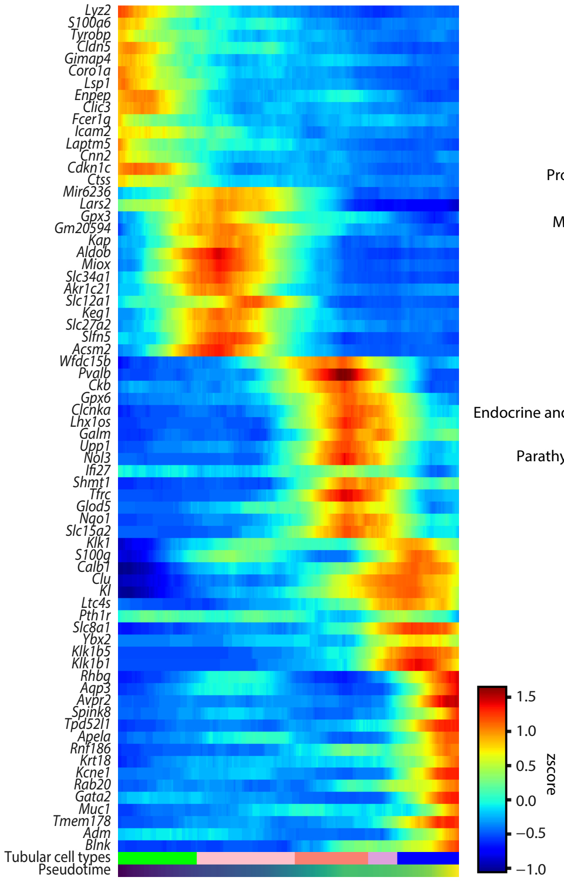

b

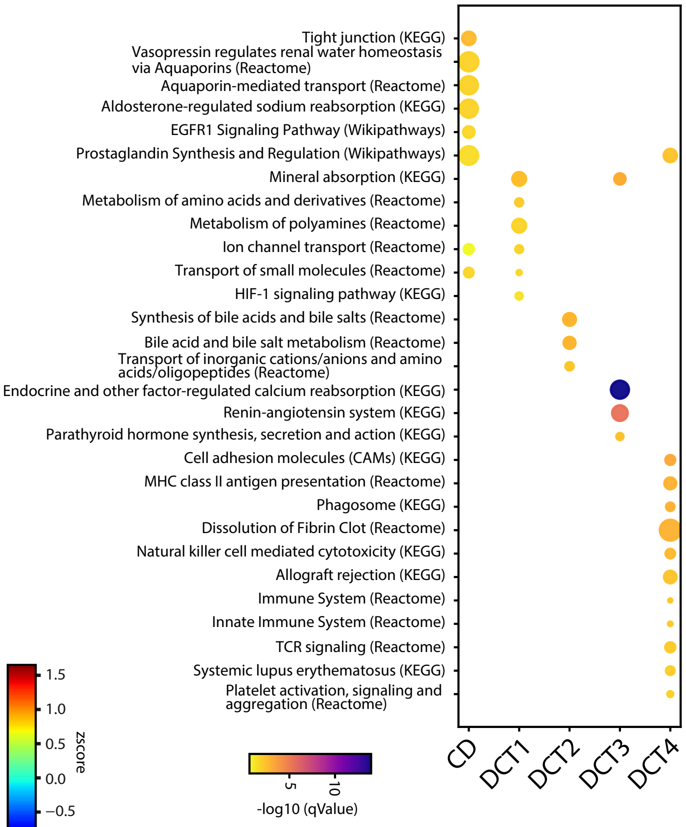

Supplementary Figure 16. The zonation and signatures of DCT subpopulations. **a** Heatmap showing top 15 genes significantly upregulated in each DCT subcluster and collecting duct cell group (CD). Cells were ordered by their zonation path and pseudotime estimates. **b** Top enriched pathways in each DCT subcluster and CD based on overexpressed genes in each cell group from differential expression analysis between DCT subclusters and CD. The pathways were selected based on their relevance to kidney tubular functions. The full list of enriched pathways is shown in Table S12. The dot colour indicates the pathway significance based on -log10(qValue) from FDR multiple testing. The dot size shows the percentage of genes in each pathway gene set were significantly upregulated in each cell group, in which the smallest and largest dot on the plot represent the percentage of 0.7 and 20 respectively.

Supplementary Figure 17

a

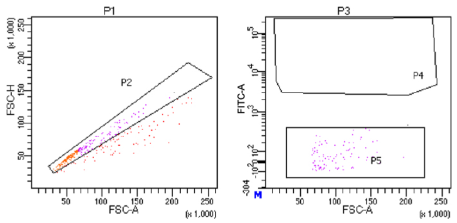

Unstained: mouse glomerular cells

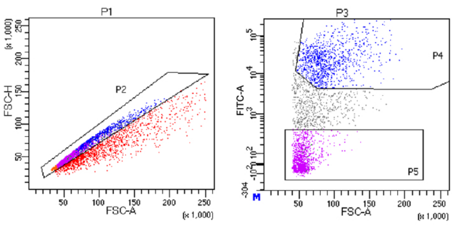

Stained: mouse glomerular cells

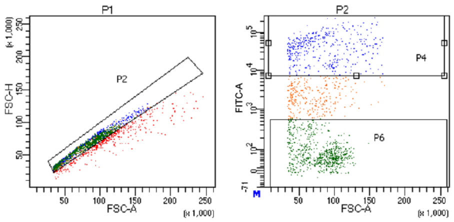

Stained: human glomerular cells

b

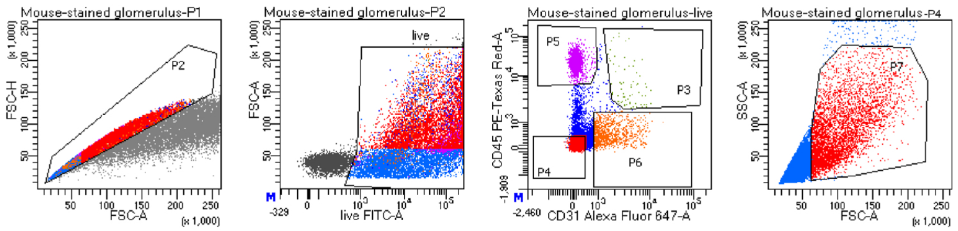

Stained: mouse glomerular cells

c

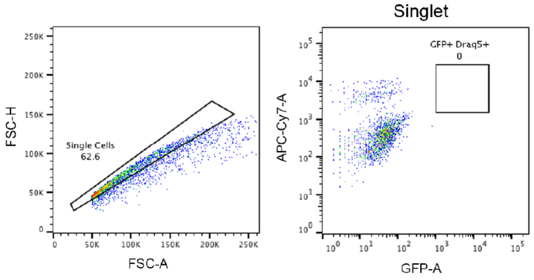

wt mouse tubular cells

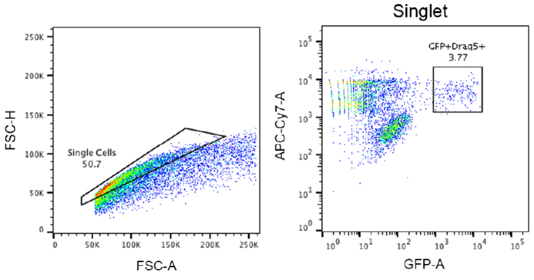

Pdgfrb-EGFP mouse glom cells

d

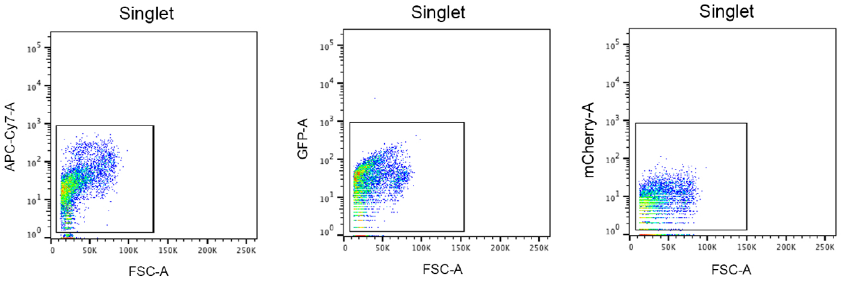

Unstained: wt mouse tubular cells

Supplementary Figure 17. FACS gating strategy for single cell sorting. **a** Unbiased sorting. Mouse and human viable single cells labelled with the live cell dye (CMFDA-Green, FITC channel) were gated to P4 for sorting. Unstained cells were used as a baseline control (left panel). **b** Enriched glomerular cell sorting. Mouse glomerular live $CD45^-$ $CD31^-$ single cells were gated to P4 followed by gating to P7 with high FSC-A for sorting. Two conjugated antibodies (anti-CD45-PE-CF594 and anti-CD31-APC) were used for cell staining. **c** $EGFP^+$ cell sorting from *Pdgfrb*-EGFP reporter mice. Wild-type mouse kidney tubular cells were used for determining gating baseline of EGFP (left panel). Viable glomerular $DRAQ5^+$ $EGFP^+$ single cells were gated for sorting (right panel). **d** The autofluorescent baseline for live cell dye DRAQ5 (APC-Cy7 channel), EGFP (GFP channel) and conjugated beads (mCherry channel) in wt mouse tubular cells.

Supplementary Table 1. Antibodies used in the manuscript

Primary antibodies

Antigen	Host	Reactivity	Isotype	Source	Cat. No.	Dilution	Application
WT1	Rabbit	H, M	mAb IgG	Abcam	ab89901	1:100	IHC-P
GATA3	Rabbit	H, M	mAb IgG	Cell Signaling	5852	1:500	IF
COL6A1	Rabbit	H, M, R	mAb IgG	Thermo Fisher	MA5-32412	1:200_h 1:400_m	IF
PDGFRB	Goat	M	Poly IgG	R&D systems	AF1042	1:200	IF
PDGFRB	Mouse	H, M	mAb IgG1	R&D systems	MAB1263	1:200	IF
aSMA-Cy3	Mouse	H, M, R	mAb IgG1	Sigma Aldrich	C6198	1:1000	IF
Calponin-1	Rabbit	H, M, R	mAb IgG	Abcam	Ab46794	1:300	IF
KDR	Rabbit	H, M	mAb IgG	Cell Signaling	2479	1:200	IF
CD31 Alexa-488	Rat	M	mAb IgG2a	Biolegend	102413	1:500	IF
CD31 APC	Rat	M	mAb IgG1	Miltenyi	130-111-541	1:25	Flow Cyt
CD45 PE-CF594	Rat	M	mAb IgG2b	BD	562420	1:1000	Flow Cyt
Desmin	Rabbit	H, M, R	Poly IgG	Abcam	ab15200	1:200	IF
LRP1	Rabbit	H, M, R	mAb IgG	Thermo Fisher	MA5-31959	1:300	IF
nNOS/NOS1	Goat	H, M, R	Poly IgG	Abcam	ab1376	1:50	IF
NT5C1A	Rabbit	H, M	Poly IgG	Sigma Aldrich	HPA050283	1:50	IF

SLC29A1	Rabbit	H, M, R	mAb IgG	Abcam	ab223851	1:50	IF
NT5C1A	Rabbit	H	Poly IgG	Sigma Aldrich	HPA054158	1:50	IF
SLC29A1	Mouse	H	mAb IgG2a	Santa cruz	sc-377283	1:50	IF
NFASC	Rabbit	H, M, R	Poly IgG	Abcam	ab31457	1:500	WB

Secondary antibodies

Host	Reactivity	Isotype	Conjugation	Source	Cat. No.	Dilution	Application
Goat	Rabbit	IgG (H+L)	Biotin	Vector Labs	PK-7200	1:1000	IHC-P
Donkey	Rabbit	IgG (H+L)	HRP	Thermo Fisher	31458	1:4000	WB
Donkey	Mouse	IgG (H+L)	Alexa fluor 647	Thermo Fisher	A-31571	1:200	IF
Donkey	Goat	IgG (H+L)	Alexa fluor 647	Thermo Fisher	A-21447	1:1000	IF
Donkey	Goat	IgG (H+L)	Alexa fluor 488	Thermo Fisher	A-11055	1:1000	IF
Donkey	Mouse	IgG (H+L)	Alexa fluor 488	Thermo Fisher	A32766	1:1000	IF
Donkey	Goat	IgG (H+L)	Alexa fluor 594	Thermo Fisher	A-11058	1:1000	IF
Goat	Rabbit	IgG (H+L)	Alexa fluor 594	Thermo Fisher	A-11012	1:1000	IF
Goat	Mouse	IgG (H+L)	Alexa fluor 488	Thermo Fisher	A11001	1:3000	IF
Goat	Rabbit	IgG (H+L)	Alexa fluor 488	Thermo Fisher	A-11008	1:1000	IF

Supplementary Table 2. PCR primers used in the manuscript

Gene Name	Species	Forward	Reverse
GAPDH	Human	5´-CATGAGAAGTATGACAACAGCCT	5´-AGTCCTTCCACGATACCAAAGT
GAPDH	Monkey	5´-CTCCTGTTCGAGAGTCAGCC	5´-TTCCCGTTCTCAGCCTTCAC
Gapdh	Pig	5´-TCGGAGTGAACGGATTTGGC	5´-TGACAAGCTTCCCGTTCTCC
Gapdh	Rat	5´-AGTGCCAGCCTCGTCTCATA	5´-ACCAGCTTCCCATTCTCAGC
Gapdh	Mouse	5´-TGTTCCTACCCCCAATGTGT	5´-TGTGAGGGAGATGCTCAGT
NPHS1	Human	5´-TGGCGATTCCTGCCTCCGTT	5´-TTCTGCTGGGAGCCCTCGTT
NPHS1	Monkey	5´-ACCGCAGAGCTCGGATAATG	5´-GCTGGCCATTCTTCAGCCA
Nphs1	Pig	5´-ATCACCTTTCTCCGCAGTGG	5´-CACTCAATGACAGGGGGTCC
Nphs1	Rat	5´-CTACAGCCCTGGAGAAGATGTG	5´-GCAGAGCTGGAATGACAGTGA
Nphs1	Mouse	5´-CTTTTGGCTTCGCTGTCACC	5´-AAGGCCCAGATTGCATCGTA
NFASC	Human	5´-AACGCCTTTGTCAGTGTGCT	5´-TAGTTGCCACCATCCAGGTT
NFASC	Monkey	5´-TTGGGACGCTGGAGTTTACC	5´-AATTTCGATGGCTCCGCCAA
Nfasc	Pig	5´-CCAACCCCTCGGATGTGAAG	5´-GAGCTGGGTGGTTAGACGG
Nfasc	Rat	5´-TCCCCCGTCATCTTCTGGA	5´-CAACTCCTCGGGTGGTGAG

Nfasc	Mouse	5´-TCTCCTGGCTGAAGGACGAT	5´-GGTCTAGCATTGCTTCAAGATGG
RXFP1	Human	5´-TCAGTCGAATTTCCCCACCA	5´-TCAGGTAAACGGGTGAGGAC
RXFP1	Monkey	5´-TGTCAACACATGCCGAGACT	5´-AGGAAAGATTCAATTCATCCAGTTT
