# Supplementary material for: Single-cell RNA sequencing reveals the mesangial identity and species diversity of glomerular cell transcriptomes
Source: Nat Commun. 2021 Apr 9;12:2141. doi: 10.1038/s41467-021-22331-9 (PMC8035407; doi:10.1038/s41467-021-22331-9)
Supplement: Supplementary file 3 — Description of Additional Supplementary Files [file 41467_2021_22331_MOESM3_ESM.pdf]

Description of Additional Supplementary Files

File Name: Supplementary Data 1
Description: Differentially expressed genes between EC 1 (glomerular ECs) and EC 2 (extra glomerular ECs).

File Name: Supplementary Data 2
Description: Over-representative pathways of genes up-regulated in glomerular endothelial cells (EC 1) compared to extra glomerular endothelial cells (EC 2).

File Name: Supplementary Data 3
Description: Over-representative pathways of genes up-regulated in extra glomerular endothelial cells (EC 2) compared to glomerular endothelial cells (EC 1).

File Name: Supplementary Data 4
Description: Genes differentially expressed between mouse MLC subclusters.

File Name: Supplementary Data 5
Description: Genes differentially expressed among mouse MLC-C2, MLC-C3 and MLC-C4.

File Name: Supplementary Data 6
Description: Genes differentially expressed between mouse EMC (MLC-C1) and MC (MLC-C4).

File Name: Supplementary Data 7
Description: Over-representative KEGG pathways of genes up-regulated in mouse mesangial cells compared to other MLC subclusters.

File Name: Supplementary Data 8
Description: Genes conservatively expressed (median log2-RPKM ≥ 4) in human and mouse glomerular mesangial cells.

File Name: Supplementary Data 9
Description: Genes conservatively expressed (median log2-RPKM ≥ 4) in human and mouse podocytes.

File Name: Supplementary Data 10
Description: Genes conservatively expressed (median log2-RPKM ≥ 4) in human and mouse GECs.

File Name: Supplementary Data 11
Description: Genes differentially expressed between distal convoluted tubule cell subclusters and collecting duct cells.

File Name: Supplementary Data 12
Description: Over-representative pathways of genes up-regulated in each DCT subcluster and collecting duct cell group.
