# Supplementary material for: Single-cell RNA sequencing reveals the mesangial identity and species diversity of glomerular cell transcriptomes
Source: Nat Commun. 2021 Apr 9;12:2141. doi: 10.1038/s41467-021-22331-9 (PMC8035407; doi:10.1038/s41467-021-22331-9)
Supplement: Supplementary file 16 — Reporting Summary [file 41467_2021_22331_MOESM16_ESM.pdf]

nature research

Corresponding author(s): Jaakko Patrakka

Last updated by author(s): Jan 5, 2021

Reporting Summary

Nature Research wishes to improve the reproducibility of the work that we publish. This form provides structure for consistency and transparency in reporting. For further information on Nature Research policies, see our Editorial Policies and the Editorial Policy Checklist.

Statistics

For all statistical analyses, confirm that the following items are present in the figure legend, table legend, main text, or Methods section.

n/a	Confirmed	
☐	☒	The exact sample size (n) for each experimental group/condition, given as a discrete number and unit of measurement
☐	☒	A statement on whether measurements were taken from distinct samples or whether the same sample was measured repeatedly
☐	☒	The statistical test(s) used AND whether they are one- or two-sided<br>*Only common tests should be described solely by name; describe more complex techniques in the Methods section.*
☐	☒	A description of all covariates tested
☐	☒	A description of any assumptions or corrections, such as tests of normality and adjustment for multiple comparisons
☒	☐	A full description of the statistical parameters including central tendency (e.g. means) or other basic estimates (e.g. regression coefficient) AND variation (e.g. standard deviation) or associated estimates of uncertainty (e.g. confidence intervals)
☐	☒	For null hypothesis testing, the test statistic (e.g. F, t, r) with confidence intervals, effect sizes, degrees of freedom and P value noted<br>*Give P values as exact values whenever suitable.*
☒	☐	For Bayesian analysis, information on the choice of priors and Markov chain Monte Carlo settings
☒	☐	For hierarchical and complex designs, identification of the appropriate level for tests and full reporting of outcomes
☒	☐	Estimates of effect sizes (e.g. Cohen's d, Pearson's r), indicating how they were calculated

Our web collection on statistics for biologists contains articles on many of the points above.

Software and code

Policy information about availability of computer code

Data collection	BCL file from Illumina HiSeq3000 were demultiplexed into fastq files using bcl2fastq software (v1.8.4). Reads were aligned to human genome hg38 by STAR aligner (v2.4.2). RNA-seq gene expression were calculated by rpkmforgenes. Data analysis were performed with R 3.6.3 and Python 3.6.5. Integration of immune cells from our study with public kidney immune cell atlas was done by using Seurat (v3.2.2)package. The soft-argmax algorithm used in our prediction was from numpy 1.15. The softwares BD FACSDiva v8.0.2 in BD Aria-II and BD FACSChorus in FACSMelody v1.1 were used for FACS data collection and cell sorting.
Data analysis	We have described relevant softwares used in each analysis in the methods section. The analysis code are deposited in GitHub and can be available upon request. The software FlowJo v10 was used for FACS data analysis of phagocytosis assays.

For manuscripts utilizing custom algorithms or software that are central to the research but not yet described in published literature, software must be made available to editors and reviewers. We strongly encourage code deposition in a community repository (e.g. GitHub). See the Nature Research guidelines for submitting code & software for further information.

Data

Policy information about availability of data

All manuscripts must include a data availability statement. This statement should provide the following information, where applicable:

- Accession codes, unique identifiers, or web links for publicly available datasets
- A list of figures that have associated raw data
- A description of any restrictions on data availability

Raw sequencing data of mouse Smart-seq2 scRNA-seq together with processed data of human and mouse used in this study has been deposited in Gene Expression Omnibus (GEO) with the accession number of GSE160048. Human Smart-seq2 scRNA-seq raw data has been submitted to European Genome-Phenome Archive

(EGA) with the accession number EGAS00001004943. In addition, a public searchable database of our kidney glomerular single cell atlas can be accessed via https://patrakkalab.se/kidney.

Field-specific reporting

Please select the one below that is the best fit for your research. If you are not sure, read the appropriate sections before making your selection.

☒ Life sciences ☐ Behavioural & social sciences ☐ Ecological, evolutionary & environmental sciences

For a reference copy of the document with all sections, see nature.com/documents/nr-reporting-summary-flat.pdf

Life sciences study design

All studies must disclose on these points even when the disclosure is negative.

Sample size	This single-cell RNA-seq study was designed to generate single cell transcriptomes from the mouse and human kidney glomerulus using the Smart-seq2 platform. This type of study highly depends on results of cell viability, RNA quality and final data analysis. An ideal result was to capture all cell types of the glomerulus and each cell type should include > 10 cells robust for classification. Total of 4344 cells from mouse and 766 cells from human kidneys passed the quality control. Data analysis demonstrated that cell population clusters based on these cells cover all glomerular cell types and glomerulus-associated cell populations. Thus, the sample size is sufficient to reach our aim.
Data exclusions	Based on previous studies, we set up the criteria of good quality cells: 1) ≥ 50,000 sequence reads; 2) ≥ 40% of reads uniquely aligned to the genome; 3) ≥ 40% of these reads mapping to RefSeq annotated exons; 4) < 10% of uniquely mapped reads from ERCC spike-ins; and 5) ≥ 500 genes with RPKM ≥ 1. Cells failed to meet these criteria were excluded from subsequent analysis. In addition, cells predicted to be doublets were also removed.
Replication	The gene expression profiles reported herein are generated from dozens to thousands of individual cells that were collected from 26 individual animals and 8 human biopsies at different time points. All experimental findings identified by single cell transcriptomes were reliably and successfully reproduced by independent datasets and validated using experimental assays. All experiments were independently performed. For validation, immunostaining, RT-PCR and Western blots were carried out.
Randomization	Animals were selected based on sex (male), age (10 - 20 weeks old) and genotype (for cell sorting of fluorescent reporter expression). For only antibody based cell sorting random C57Bl6/J male mice at corresponding age from the core breeding were selected. All other criteria were not considered and as such, randomized. Human samples were obtained based on fully randomized assignment by the coordinator committee. To capture all cell types of the glomerulus, we randomly sort all viable cells.
Blinding	For scRNA-seq studies, researchers were blinded to group allocation. For example, cell preparation from mouse and human kidney was performed by experienced investigators with biomedical background. Coded samples were sequenced by the single cell sequencing core facility. Sequencing data was finally analyzed by a bioinformatician independent of the kidney research group and absent of kidney knowledge. For validation experiments, the same principle was followed, where at least two researchers were blindly assigned to either lab work or data analysis. For example, one performed immunostaining and the other performed confocal imaging.

Reporting for specific materials, systems and methods

We require information from authors about some types of materials, experimental systems and methods used in many studies. Here, indicate whether each material, system or method listed is relevant to your study. If you are not sure if a list item applies to your research, read the appropriate section before selecting a response.

Materials & experimental systems

n/a	Involved in the study	
☐	☒	Antibodies
☒	☐	Eukaryotic cell lines
☒	☐	Palaeontology and archaeology
☐	☒	Animals and other organisms
☐	☒	Human research participants
☒	☐	Clinical data
☒	☐	Dual use research of concern

Methods

n/a	Involved in the study	
☒	☐	ChIP-seq
☐	☒	Flow cytometry
☒	☐	MRI-based neuroimaging

Antibodies

Antibodies used	Information of all primary and secondary antibodies used in the study are presented in supplementary table 1. WT1 Abcam ab89901 clone #CAN-R9(IHC)-56-2; GATA3 Cell signaling Tech 5852 clone #D13C9; COL6A1 Thermo Fisher MA5-32412 clone #SD83-03; PDGFRB R&D systems AF1042; PDGFRB R&D systems MAB1263 clone #PR7212; aSMA-Cy3 Sigma Aldrich C6198 clone #1A4; Calponin-1 Abcam Ab46794; KDR Cell Signaling Tech 2479 clone #55B11; CD31 Alexa-488 Biolegend 102413 clone #390; CD31 APC Miltenyi 130-111-541 clone #REA784; CD45 PE-CF594 BD Biosciences 562420 clone #30-F11; Desmin Abcam ab15200; LRP1 Thermo Fisher MA5-31959; NFASC Abcam ab31457; NOS1 Abcam ab1376; NT5C1A Sigma-Aldrich HPA050283; NT5C1A Sigma-

	Aldrich HPA054158; SLC29A1 Abcam ab223851; SLC29A1 Santa cruz sc-377283; B-actin Abcam ab8277; Biotinylated goat anti-rabbit secondary ab Vector Labs PK-7200; HRP-conjugated donkey anti-rabbit secondary ab Thermo Fisher 31458; Alexa fluor 647 donkey anti-mouse IgG (H+L) secondary ab Thermo Fisher A-31571; Alexa fluor 647 donkey anti-goat IgG (H+L) secondary ab Thermo Fisher A-21447; Alexa fluor 488 donkey anti-goat IgG (H+L) secondary ab Thermo Fisher A-11055; Alexa fluor 488 donkey anti-mouse IgG (H+L) secondary ab Thermo Fisher A32766; Alexa fluor 594 donkey anti-goat IgG (H+L) secondary ab Thermo Fisher A-11058; Alexa fluor 594 goat anti-rabbit IgG (H+L) secondary ab Thermo Fisher A-11012; Alexa fluor 488 goat anti-rabbit IgG (H+L) secondary ab Thermo Fisher A-11008; Alexa fluor 488 goat anti-mouse IgG (H+L) secondary ab Thermo Fisher A-11001
Validation	The specifics including validation and dilution on each antibody used in the study are also summarized in supplementary table 13. Further information about antibody specificity, species cross-reactivity, recommended applications and links to relevant publications can be found on the manufacturers' website.<br>WT1 human/mouse; IHC validated by the manufacturer Abcam (1:300)<br>GATA3 human/mouse; IF validated by the manufacturer CST (1:1600)<br>COL6A1 human/mouse/rat; IHC-P validated by the manufacturer Thermo Fisher (1:100-500)<br>PDGFRB mouse; IHC validated by the manufacturer R&D systems(1:10-40); IF not validated by the manufacturer<br>PDGFRB human; IF validated by using flow cytometry (Stem Cells 2007;25:1737)<br>αSMA-Cy3 human/mouse/rat; IF validated by the manufacturer Sigma Aldrich and users (Sci Rep 2018;8:430)<br>Calponin-1 human/mouse/rat; IF validated by the manufacturer Abcam (1:500)<br>KDR human/mouse; IF validated by the manufacturer Cell Signaling Tech (1:200)<br>CD31 Alexa-488 mouse; IF validated by the manufacturer Biolegend (1:250)<br>CD31 APC mouse; Flow cytometry validated by the manufacturer Miltenyi (1:50)<br>CD45 PE-CF594 mouse; Flow cytometry validated by the manufacturer BD Biosciences and users (Cell Metab 2018;27:588)<br>Desmin human/mouse/rat; IF validated by the manufacturer Abcam (1:100) (Nat Comm 2019;10:2817)<br>LRP1 human/mouse/rat; IF validated by the manufacturer Thermo Fisher (1:200)<br>NFASC human/mouse/rat; WB validated by the manufacturer Abcam (1:500)<br>NOS1 human/mouse/rat; IF validated by the manufacturer Abcam (1:50)<br>NT5C1A (HPA050283) human/mouse; IF validated by the manufacturer Sigma-Aldrich and the user (1:50)<br>NT5C1A (HPA054158) human; IF validated by the manufacturer Sigma-Aldrich (1:50)<br>SLC29A1 (Abcam ab223851) human/mouse/rat; IF validated by the manufacturer Abcam and the user (1:50)<br>SLC29A1 (sc-377283) human/mouse/rat; IF validated by the manufacturer Santa cruz (1:50)<br>B-actin (ab8277) human/mouse/rat; WB validated by the manufacture Abcam (1:1000)<br>Biotinylated goat anti-rabbit secondary ab (PK-7200) IHC validated by the manufacturer Vector Labs (1:1000)<br>HRP-conjugated donkey anti-rabbit IgG (H+L) secondary ab (31458) WB validated by the manufacturer Thermo Fisher 1:4000<br>Alexa fluor 647 donkey anti-mouse IgG (H+L) secondary ab (A-31571) IF validated by the user for STED imaging (1:200)<br>Alexa fluor 647 donkey anti-goat IgG (H+L) secondary ab (A-21447) IF validated by the manufacturer Thermo Fisher (1:1000)<br>Alexa fluor 488 donkey anti-goat IgG (H+L) secondary ab (A-11055) IF validated by the manufacturer Thermo Fisher (1:1000)<br>Alexa fluor 488 donkey anti-mouse IgG (H+L) secondary ab (A32766) IF validated by the manufacturer Thermo Fisher (1:1000)<br>Alexa fluor 594 donkey anti-goat IgG (H+L) secondary ab (A-11058) IF validated by the manufacturer Thermo Fisher (1:1000)<br>Alexa fluor 594 goat anti-rabbit IgG (H+L) secondary ab (A-11012) IF validated by the manufacturer Thermo Fisher (1:1000)<br>Alexa fluor 488 goat anti-rabbit IgG (H+L) secondary ab (A-11008) IF validated by the manufacturer Thermo Fisher (1:1000)<br>Alexa fluor 488 goat anti-mouse IgG (H+L) secondary ab (A-11001) IF validated by the manufacturer Thermo Fisher (1:1000)

Animals and other organisms

Policy information about studies involving animals; ARRIVE guidelines recommended for reporting animal research

Laboratory animals	Wild-type male C57BL/6J mice with age of 12.6±2.6 weeks old; Wild-type male tdTomato mice with mixed background, generated by crossing the Pod-Cre female mice with the floxed STOP tdTomato reporter male mice, with age of 16 weeks old; Pdgfrb-EGFP+/- male mice with age of 12 weeks old. All animals were housed in standard, single ventilated cages with 12 hr light-12 hr dark cycle, and had ad libitum access to water and chow. The house temperature was maintained as 20 ± 2°C and the relative humidity was kept as 50 ± 5%.
Wild animals	This study did not involve wild animals.
Field-collected samples	This study did not involve samples collected from the field.
Ethics oversight	For mouse work, all experimental protocols were approved by The Linköping Ethical Committee for Research Animals ("Linköpings djurförsöksetiska nämnd"), Linköping, Sweden (archive number DNR 41-15).

Note that full information on the approval of the study protocol must also be provided in the manuscript.

Human research participants

Policy information about studies involving human research participants

Population characteristics	Total of 8 donor kidney biopsies were obtained in this study. They were healthy Swedish citizens (3 male and 5 female with the average age of 48.2±12 years old). Information of genotyping is unavailable.
Recruitment	All participants were volunteers donating a kidney to a patient with end-stage renal disease. All gave written consent to use biopsies for research. We received renal biopsies randomly from these individuals and all donors were healthy. Thus, no selection bias could have occurred.
Ethics oversight	The ethical committee, Regionala etikprövningsnämnden i Stockholm, approved the biopsy protocol (2010/579-31,

Ethics oversight	2016/615-32) and all donors gave informed consent to use the biopsies for the study. Detailed information is available at Etikprovningsmyndigheten (www.etikprovningsmyndigheten.se).

Note that full information on the approval of the study protocol must also be provided in the manuscript.

Flow Cytometry

Plots

Confirm that:

☒ The axis labels state the marker and fluorochrome used (e.g. CD4-FITC).

☒ The axis scales are clearly visible. Include numbers along axes only for bottom left plot of group (a 'group' is an analysis of identical markers).

☒ All plots are contour plots with outliers or pseudocolor plots.

☒ A numerical value for number of cells or percentage (with statistics) is provided.

Methodology

Sample preparation	A detailed description of the cell isolation and sample preparation from animals and human biopsies can be found in the method section of the manuscript.<br>Isolated mouse or human glomeruli were dissociated using type IV collagenase (1 mg/ml) and pronase (1 mg/ml) at 37°C for 15 min with shaking. After wash, cells were suspended in sorting buffer (PBS no Ca++/Mg++ containing 1 mM EDTA and 1% FCS). A live cell dye (CMFDA-Green) was added (1:1000) before sorting. For immunostaining, two conjugated antibodies, CD31-APC (1:50) and CD45-PE-CF594 (1:1000) were added in cell suspension in 50 µl sorting buffer at 4°C for 15 min. After wash, cells were resuspended in sorting buffer. The live cell dye (1:1000) was added before sorting. For phagocytosis assay using FACS, prepared glomerular cells from Pdgfrb-EGFP mice were incubated with latex beads conjugated with the pH-sensitive fluorescence-Red at 37°C for 30 min and as a baseline control, cells were incubated with the beads at 4°C for 30 min.
Instrument	Two FACS sorter, BD Aria II or BD FACSMelody, from BD Biosciences were used.
Software	The softwares used in this study are BD FACSDiva 8.0.2 for BD Aria-II, BD FACSChorus for FACSMelody and FlowJo v10 for data analysis.
Cell population abundance	For unbiased sorting, we sorted all viable cells determined by using a live cell dye, CellTracker CMFDA-Green (Ex492/Em517 ThermoFisher). Compared with unstained cells, only viable cells positive for the live cell dye were sorted, accounting for >80% out of all single cells. For cell enrichment by using biased sorting, we excluded leukocytes and endothelial cells determined by staining two conjugated antibodies CD31 and CD45. Live cell dye+ viable single cells positive for CD31 and CD45 were gated, accounting for 45%. Among them, relative big cells further gated for sorting were about 31%. To sort a specific PDGFRB+ cell population as an independent replication, we gated this cell type using the Pdgfrb-EGFP reporter mouse line and observed its abundance with 3.8% out of viable single cells in isolated mouse glomerular cells.
Gating strategy	Four gating strategies for specific aims were designed in this study. Strategy A: (1) P1, using FSC-A/SSC-A to remove debris/very small events; (2) P2, using FSC-H/FSC-A to gate single cells within the setting range; (3) P4, using GFP-A or FITC-A/FSC-A to gate events positive for the live cell dye (CellTracker CMFDA-Green). We sorted randomly all P4 cells. Strategy B: To enrich Pdgfrb+ cells, we excluded leukocytes and endothelial cells through staining of antibodies CD31-APC, CD45-PE-CF594 (BD Biosciences), together with CMFDA-Green. live cells (P live) negative for CD31 and CD45 were gated to P4 using CD45 PE-Texas Red-A/CD31 Alexa 647-A. Then relatively big cells were gated to P7 using SSC-A/FSC-A for final sorting. Strategy C: To sort EGFP-labeled PDGFRB+ cells, (1) P1, using FSC-A/SSC-A to remove debris/very small events; (2) P2, using FSC-H/FSC-A to gate single cells (singlet); (3) we gated cells positive for GFP (GFP-A) and DRAQ5 (APC-Cy7-A, the viable cell dye) for sorting. Strategy D: (1) P1, using FSC-A/SSC-A to remove debris/very small events; (2) P2, using FSC-H/FSC-A to gate single cells (singlet); (3) P3, using APC-Cy7-A/FSC-A to gate viable cells; (4) we gated cells positive for mCherry (mCherry-A, uptake of beads) and GFP (GFP-A) for PDGFRB+ bead+ cells. Representative FACS gating plots for cell sorting are presented in supplementary Figure 16a-c. All original FACS gating plots and raw data for phagocytosis assays in mouse kidney (n=3) and blood leukocytes (n=3) are presented in the Source data file.

☒ Tick this box to confirm that a figure exemplifying the gating strategy is provided in the Supplementary Information.
